# Enhanced Stilbene Production and Excretion in *Vitis vinifera* cv Pinot Noir Hairy Root Cultures

**DOI:** 10.3390/molecules21121703

**Published:** 2016-12-10

**Authors:** Leo-Paul Tisserant, Aziz Aziz, Nathalie Jullian, Philippe Jeandet, Christophe Clément, Eric Courot, Michèle Boitel-Conti

**Affiliations:** 1 Laboratoire de Biologie des Plantes et Innovation EA 3900, SFR Condorcet FR CNRS 3417, UFR des Sciences, Ilot des Poulies, Université de Picardie Jules Verne, 33 rue Saint Leu, 80039 Amiens Cedex, France; leo-paul.tisserant@univ-reims.fr (L.-P.T.); nathalie.pawlicki@u-picardie.fr (N.J.); 2 Unité de Recherche Vignes et Vins de Champagne EA 4707, SFR Condorcet FR CNRS 3417, UFR des Sciences Exactes et Naturelles, Université de Reims Champagne-Ardenne, BP 1039, 51687 Reims Cedex 2, France; aziz.aziz@univ-reims.fr (A.A.); christophe.clement@univ-reims.fr (C.C.); eric.courot@univ-reims.fr (E.C.)

**Keywords:** *Vitis vinifera*, hairy roots, resveratrol, viniferins, methyl jasmonate, cyclodextrins

## Abstract

Stilbenes are defense molecules produced by grapevine in response to stresses including various elicitors and signal molecules. Together with their prominent role in planta, stilbenes have been the center of much attention in recent decades due to their pharmaceutical properties. With the aim of setting up a cost-effective and high purity production of resveratrol derivatives, hairy root lines were established from *Vitis vinifera* cv Pinot Noir 40024 to study the organ-specific production of various stilbenes. Biomass increase and stilbene production by roots were monitored during flask experiments. Although there was a constitutive production of stilbenes in roots, an induction of stilbene synthesis by methyl jasmonate (MeJA) after 18 days of growth led to further accumulation of ε-viniferin, δ-viniferin, resveratrol and piceid. The use of 100 µM MeJA after 18 days of culture in the presence of methyl-β-cyclodextrins (MCDs) improved production levels, which reached 1034 µg/g fresh weight (FW) in roots and 165 mg/L in the extracellular medium, corresponding to five-and 570-fold increase in comparison to control. Whereas a low level of stilbene excretion was measured in controls, addition of MeJA induced excretion of up to 37% of total stilbenes. The use of MCDs increased the excretion phenomenon even more, reaching up to 98%. Our results demonstrate the ability of grapevine hairy roots to produce various stilbenes. This production was significantly improved in response to elicitation by methyl jasmonate and/or MCDs. This supports the interest of using hairy roots as a potentially valuable system for producing resveratrol derivatives.

## 1. Introduction

Stilbenes have been the center of much attention over recent decades due to their valuable biological properties. The most studied stilbene, resveratrol, has been described as an important plant defense molecule, with antifungal and antibacterial activities [1,2,3,4]. Although stilbenes are found in grapevines at levels considerably lower than flavonoid-type compounds, they possess outstanding pharmaceutical properties [5] as antioxidants [6] as well as anticancer agents [7,8]. Resveratrol derivatives such as viniferins also show an interesting therapeutic potential, mainly in cancer treatment [9,10]. The main natural sources of resveratrol available on the market result from its extraction from Japanese knotweed (*Falliopa japonica*), which produces large quantities of piceid (a β-d-resveratrol glucoside), which is then de-glycosylated to obtain resveratrol [11,12]. Consumers’ preference indeed remains in favor of stilbenes from natural sourcing. Few resveratrol derivatives, except the dehydrodimer ε-(+)-viniferin, are available and the current production of these derivatives does not yet meet the pharmaceutical market standards in terms of quantity and purity. Whereas the biosynthetic pathway of resveratrol has been very well characterized [13], those of many resveratrol derivatives are unknown. Currently, the sustainable production of resveratrol derivatives remains difficult to achieve due to their overall low concentrations in plant extracts and problems in purifying them. Alternative strategies including microorganism or plant cell cultures have been tested as valuable systems for stilbene sourcing [14]. Genetically engineered microorganisms or plants for the production of stilbenes would enable a cost-effective supply of these molecules, but the overall low yields and the still incomplete understanding of the biosynthetic pathways of complex stilbenes make their use of limited interest [15,16,17]. Moreover, there is a considerable interest in searching for sources of stilbenes without recombinant genetic modification. Grapevine cell suspensions, which can produce and excrete resveratrol into their culture medium, have led to promising results [18,19]. In grapevine cell systems in either flasks or bioreactors, resveratrol production can reach up to 7 g/L in response to elicitation treatments with various compounds such as signal molecules (jasmonate or methyl jasmonate) or chelating agents or drug carriers such as cyclodextrins (CDs) (for a review, see [18,19]).

The use of hairy roots offers the advantage of high genetic and biochemical stabilities of organ culture systems coupled with growth rates similar to those of cell suspensions [20,21], without the need for plant growth regulators. In this context, hairy roots of peanut (*Arachis hypogaea*) and Muscadine grape (*Vitis rotundifolia*) have already been developed [22,23,24]. Both of these plants have previously been described as naturally producing resveratrol and resveratrol derivatives [25]. Although stilbenes have been described in various taxonomically unrelated species, the highest molecular diversity within this chemical group has been found in the *Vitis* spp. genus [26]. Use of the sequenced cultivar *Vitis vinifera* cv Pinot Noir 40024 hairy roots thus appears a good tool for a comprehensive analysis of the organ-specific biosynthesis of stilbenes. Moreover, Pinot Noir has been reported as naturally producing high levels of stilbenes in canes [27] and berries [28].

In this study, we describe the establishment of *Vitis vinifera* cv Pinot Noir PN 40024 hairy root lines from in vitro plantlets. Various culture conditions were tested and the growth kinetics were defined in those yielding the highest growth rate. The ability of *Vitis vinifera* hairy roots to produce and excrete various stilbenes was monitored during growth kinetics experiments in response to methyl jasmonate (MeJA) or CD treatment alone or in combination.

## 2. Results and Discussion

### 2.1. Establishment of Hairy Root Lines (HRs)

As suggested by some previous results [29], the transformation of *Vitis* spp. by *R. rhizogenes* is very dependent on species, on the bacterial strain used as well as the explants tested. In this study, only the sequenced grapevine cultivar PN 40024 [30] was tested, as it represents particular interest for research purpose. Two different bacterial strains were tested to infect various explants from in vitro plantlets. The phytohormone 2,4-d was used to make plant cells more competent, as previously reported in *Arabidopsis thaliana* and *Beta vulgaris* L. [31,32].

Leaves with petioles and stem explants of plantlets of *V. vinifera* PN 40024 transformed using *Rhizobium rhizogenes* ICPBTR7, led to a rapid loss of viability of the explants and thus yielded no roots either by immersion or wounding of the explants or by direct pricking of the plantlets. The plantlets pricked with an infected needle showed no development of roots regardless of the strain used.

Only the Pinot Noir leaves and stem explants transformed with *R. rhizogenes* ATCC15834 by immersion in the bacterial suspension or by wounding with an infected scalpel yielded a particularly large amount of transformed roots. Over the 200 explants tested, several hundreds of roots developed after two weeks, mostly along the petioles and leaf veins.

**Figure 1:**
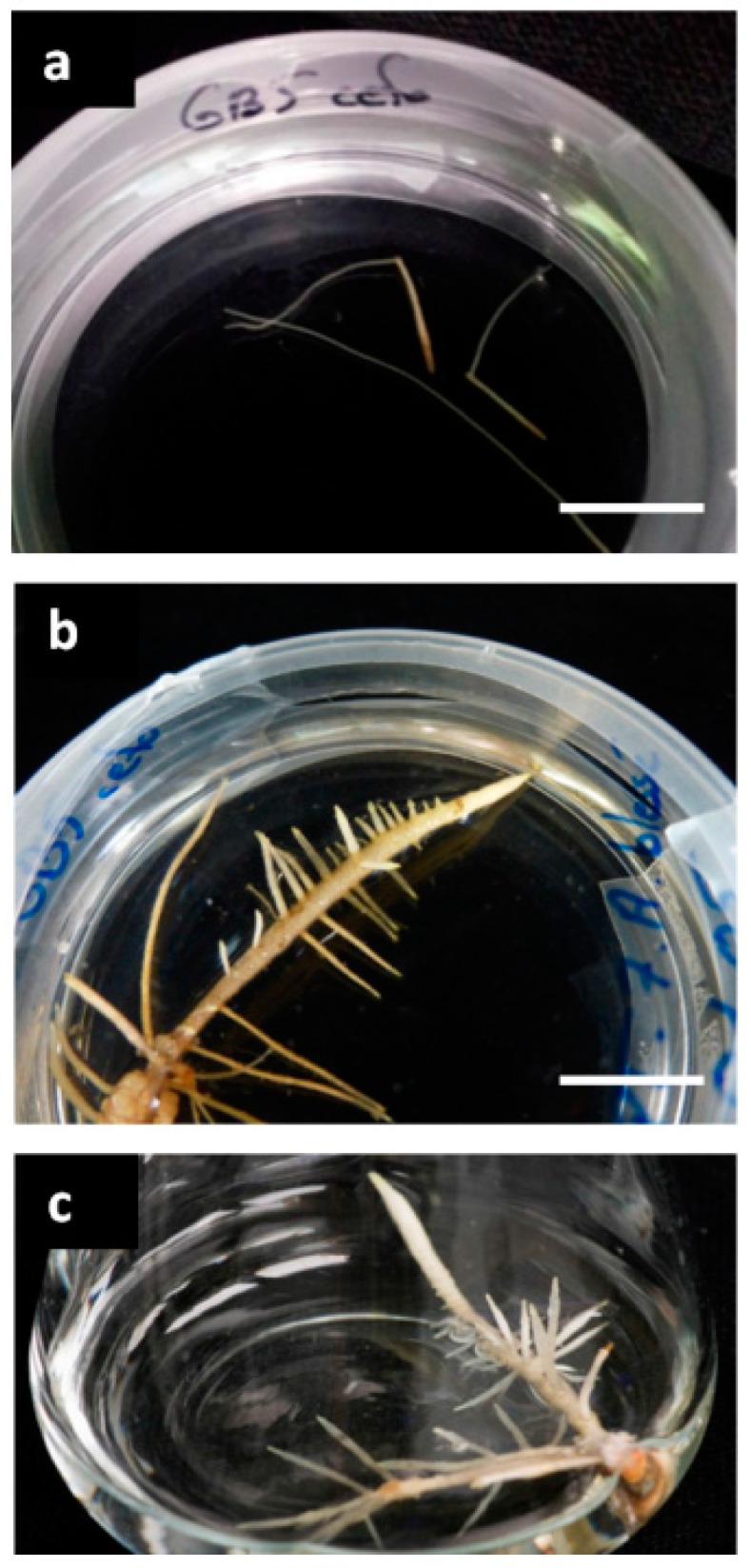
Observations of the different phenotypes of roots isolated:(**a**) roots from the first series isolated after the second subculture (28 days); (**b**) roots from the second series isolated after the second subculture (21 days of culture); and (**c**) roots from the second series after 40 days growth in 100-mL shaken flasks with 20 mL culture medium. Bars represent 1 cm.

Once isolated on solid B5 medium supplemented with 3% sucrose, these lines showed a slow growth rate during the first weeks of culture and grew thinner over time until death after two months (Figure 1a). These roots were probably adventitious roots due to the very well-known rooting ability of grapevine and as a result of the addition of 2,4-dichlorophenoxyacetic acid in the bacterial suspension used to infect the explants. A second series of roots appeared on the same Pinot Noir explants four weeks after infection with *R. rhizogenes* ATCC 15834 using the two previous methods. Once they were 1–2 cm long, roots were isolated and cultivated on solid B5 medium supplemented with 3% sucrose (B5-30). These roots showed a completely different phenotype from the first ones, with a diameter ranging from 1 to 3 mm and a higher branching rate (Figure 1a,b). This phenotype showed some similarity to that described on Muscadine grape, with thick roots, very short root hairs, and a higher branching rate (10–15 branches per 5 cm) compared to the 4–5 branches per 5 cm for Muscadine HRs, as reported in a previous work [24]. In the second series of roots that appeared, 80 different lines were isolated and cultivated on B5-30 solid medium. The 60 lines displaying the best growth rates were then transferred to a B5-30 liquid medium (Figure 1c) to be further selected on growth capacity. Nine lines were thus conserved and their transformation was verified by confirmation of the presence of transferred *rolC* gene and the absence of *virD2* bacterial virulence gene (Figure S1). As few differences in terms of phenotype, growth rate and phytochemical profile were observed between these lines (data not shown), the root line 19A was chosen as representative and used further.

### 2.2. Effects of Culture Medium and Sucrose Concentration on HR Growth

**Figure 2:**
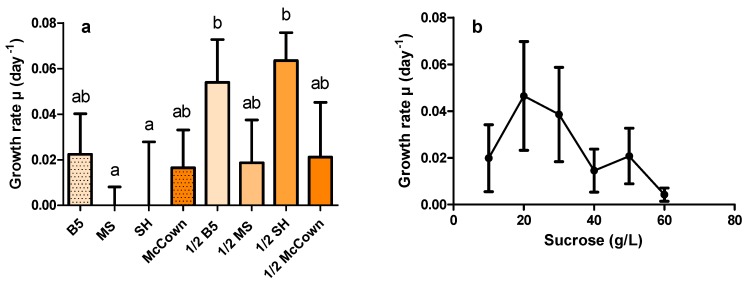
Effects of media and sucrose concentration on HR growth rate: (**a**) effect of various standard media and 1/2 dilutions, with 3% sucrose on the growth rate of line 16C; and (**b**) effect of sucrose concentrations in B5 medium on the growth of line 19A. Average growth rate µ and its standard error were calculated from weight measurements made over 35 days with renewal of culture media every three or four days. Each point is composed of three biological replicates. Statistical differences were tested using ANOVA (α = 5%). Letters above dataset point out statistically different groups.

The composition of the medium used is known to have an important impact on the growth capacity and secondary metabolite production of in vitro cultures. The observed slow growth of the selected lines strongly suggested that the medium composition could be further improved. To search for better culture conditions, four commonly used culture media, Gamborg (B5), McCown (MC), Murashige and Skoog (MS), Schenk and Hildebrandt (SH), and their half-strength dilutions, were compared. In addition, six different sucrose concentrations ranging from 1% to 6% were tested (Figure 2).

A preference for 1/2 media seems to occur regardless of the medium tested (Figure 2a). Only the B5 and MC media enabled growth when non-diluted. Amongst the 1/2 media, it would seem that the SH medium yielded the best growth rate (µ), reaching µ = 0.063 day^−1^ (doubling time, Td = 10.9 days), along with B5. Determination of the growth rate using different sucrose concentrations in the B5 medium (Figure 2b) showed that the range of 1% to 3% sucrose concentrations in the medium seems to be the most effective, reaching a growth rate of 0.046 day^−1^ (doubling time Td = 14.9 days) for 2% sucrose. As well as showing a low growth rate, the roots grown on medium with 5% and 6% sucrose started browning after three weeks, suggesting significant stress under these conditions. This effect could be caused by an osmotic stress due to high sugar concentrations [33]. Thus, 1/2 SH medium with 2% sucrose was used for further experiments.

### 2.3. Growth and Stilbene Production Kinetics

#### 2.3.1. Biomass Production

**Figure 3:**
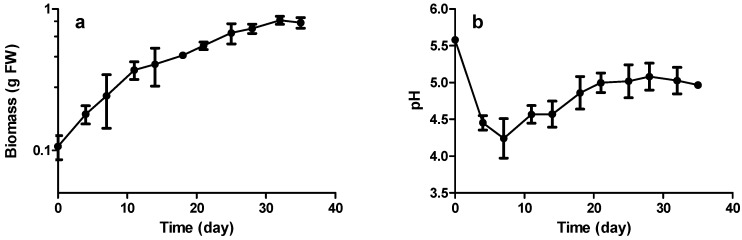
Increase in: biomass (fresh weight) (**a**); and pH (**b**) during growth kinetics. Each point represents the mean with standard deviation of three biological replicates.

The growth kinetics were evaluated using line 19A in the improved growth conditions of 1/2 SH medium with 2% sucrose (Figure 3).

The semi-log representation of the growth curve (Figure 3a) of grapevine HRs shows no significant lag phase, starting directly with an exponential growth phase up to Day 11, followed by a slowing phase until Day 31 and a stationary phase up to Day 35. The rapid diminution in growth could be due to the relatively low amounts of sucrose in the culture medium. Figure 3b displays a rapid fall in the pH during the first days of culture up to 4–7 days. The pH then rises to 5 at day 20 before stabilizing, in the phases corresponding to both the slower growth and the stationary phases. This suggests that pH can be used as a rapid, non-destructive tool to monitor growth in grapevine HR cultures.

Growth rate was calculated between Day 0 and Day 11, giving a µ value of 0.11 day^−1^ (Td = 6.2 days). In comparison, peanut and Muscadine HRs have been reported to have a doubling time of 10.2 and 10.7 days, respectively, without any addition of growth regulators [23,24].

#### 2.3.2. Constitutive Stilbene Production in Roots

**Figure 4:**
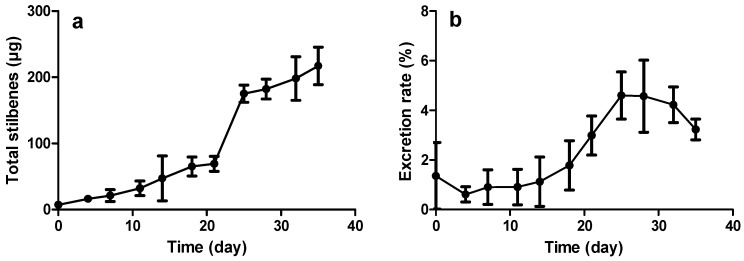
Stilbene production during growth kinetics: (**a**) total production of stilbenes (µg of stilbenes per culture flask); and (**b**) excreted stilbenes as a proportion of total stilbenes. Each point represents the mean with standard deviation of three biological replicates.

The analysis of stilbenes during the time course experiments shows that a basal production takes place in roots (Figure 4), where they probably play a role as phytoanticipins as already mentioned in natural grapevine roots as well as in the perennial parts of the plant [34].

Here, only the resveratrol glucoside, piceid, resveratrol, and the two dehydrodimers, ε-viniferin and δ-viniferin, were quantified using external standards. Further use of the term “total stilbenes” refers to the sum of all these molecules. A close relationship between the growth phase and the production of secondary metabolites in roots can be observed, especially when the metabolism tends to switch from primary to secondary at the end of the exponential growth phase. As shown in Figure 4a, the basal production of stilbenes per flask rises slowly until the end of the exponential phase at Day 21, before rapidly increasing up to 175 µg at Day 25 and 217 µg after 35 days. It is interesting to note the same trend in the proportion of excreted stilbenes (Figure 4b). The low proportion of 1%–5% excreted stilbenes vs. total stilbenes is consistent with those measured in similar systems without elicitation [23,24].

### 2.4. Induction of Stilbene Production in HRs in Response to Various Elicitors

#### 2.4.1. Elicitation with Methyl Jasmonate

Stilbene production, and particularly that of resveratrol, is known to be strongly induced by various environmental stresses, signaling molecules and drug transporters [19,35]. Such compounds have been used in various plant cell systems to mimic a stress and thus induce the production of numerous metabolites of interest [14,36,37]. Amongst them, the jasmonic acid ester, methyl jasmonate (MeJA), has been shown to induce stilbene production mainly in grapevine cell suspensions [38,39,40,41,42,43,44,45,46,47].

**Figure 5:**
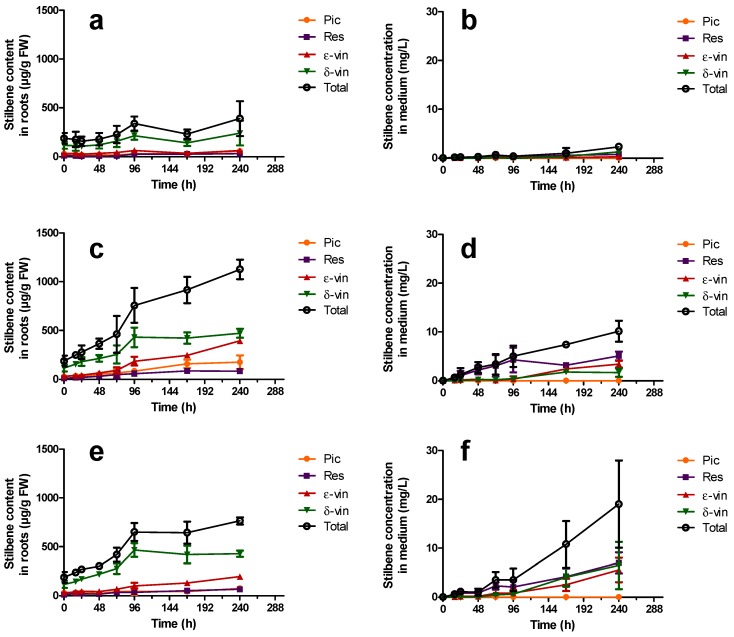
Amounts of stilbenes produced after 18 days of culture following MeJA elicitation in: HRs (**a**,**c**,**e**); and in the culture medium (**b**,**d**,**f**). (**a**,**b**) EtOH at 0.1% was used as the control; (**c**,**d**) 100 µM MeJA; and (**e**,**f**) 200 µM MeJA. Pic: piceid (●); Res: Resveratrol (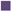); ε-vin: ε-viniferin (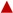); δ-vin: δ-viniferin (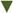); Total (○) (sum of previous stilbenes). Each point represents the mean with standard deviation of three biological replicates.

Figure 5 shows the response in terms of stilbene production in roots and in the culture medium following treatment with 100 μM MeJA up to 10 days after induction. Addition of 0.1% EtOH (*v*/*v*) (corresponding to the alcoholic content upon addition of MeJA) to the control cultures shows no significant effect of the solvent on root or medium stilbene contents. All stilbene production values in roots were expressed here as micrograms per gram of fresh weight. In order to relate the obtained data with root dry weight, we determined during replicate experiments the ratio dry root weight over fresh root weight which was in the order of 16% ± 7%. As a result, stilbene production in μg/g DW can be obtained by multiplying stilbene production in μg/g fresh weight (FW) by a factor of around 7.

Figure 5a displays a basal stilbene production in roots of around 200 µg/g FW, reaching up to 390 µg/g FW 10 days after elicitation. The low stilbene concentrations measured in the extracellular medium, reaching a maximum of 2.3 mg/L at Day 10, are consistent with those previously described [23,24]. However, application of MeJA at doses of 100 µM and 200 µM has a clear inducing effect on the stilbene contents of both the roots and the extracellular medium. Unlike described in peanut hairy roots treated with MeJA, the stilbene concentration in the culture medium did not reach its maximum after 12–48 h and no fall in concentration was observed during the tested period [22]. One hundred micromoles of MeJA (Figure 5c) gives rise to a quick increase in the stilbene root content between two and four days after induction, reaching 757 µg/g FW and followed by a steady increase up to 1126 µg/g FW after 10 days, corresponding to 2.2- and 2.9-fold increases respectively in comparison to control.

**Figure 6:**
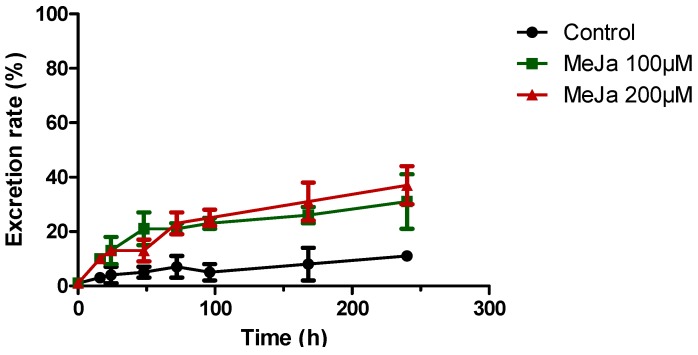
Proportion of stilbenes in the culture medium over time in control cultures (●) and cultures treated with 100 µM (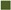) or 200 µM MeJA (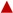). Each point represents the mean with standard deviation of three biological replicates.

In the extracellular medium (Figure 5d), 100 µM MeJA causes a similar effect, reaching stilbene contents of 5 and 10 mg/L after four and 10 days of treatment, corresponding to 12.6- and 4.3-fold increases, respectively, in comparison to control. Application of 200 µM MeJA shows a comparable effect on the total stilbene root content up to Day 4, reaching 650 µg/g FW (Figure 5e), although stabilizing afterwards. The browning of the roots observed upon treatment with 200 µM MeJA suggests a possible effect of this compound on root viability and biomass, as previously reported in *Brassica* hairy root cultures [48]. However, only the treatment with 100 µM MeJA displays statistically significant differences in biomass in comparison to the control 10 days after elicitation. Quantification of the corresponding excreted stilbenes again shows a response similar to the use of 100 µM MeJA until Day 4, reaching 3.5 mg/L. The stilbene concentration then rises to 19 mg/L at Day 10 (Figure 5f), which corresponds to 8.8- and 8.1-fold increases, respectively, in comparison to control. This observation is probably due to a release of intracellular stilbenes into the medium. All the data indicate that four days following MeJA treatment is the best timing, i.e. when induction is at a maximum and to minimize a possible detrimental effect of MeJA on root viability. For further experiments, stilbene measurements were thus carried out four days after MeJA induction. Excretion rates of stilbenes are also significantly affected by MeJA treatments (Figure 6).

While the control excretion rate rises slowly from 1% to 11% of total stilbenes in 10 days, the cultures with MeJA display excretion rates of 31% and 37% for 100 µM and 200 µM MeJA, respectively, after 10 days. No piceid was found in the medium of MeJA-treated roots and less than 10% of δ-viniferin was excreted while almost 30% ε-viniferin and 60% of resveratrol produced were found in the culture medium (Figure S2).

#### 2.4.2. Elicitation Experiments with Cyclodextrins and/or Methyl Jasmonate

**Figure 7:**
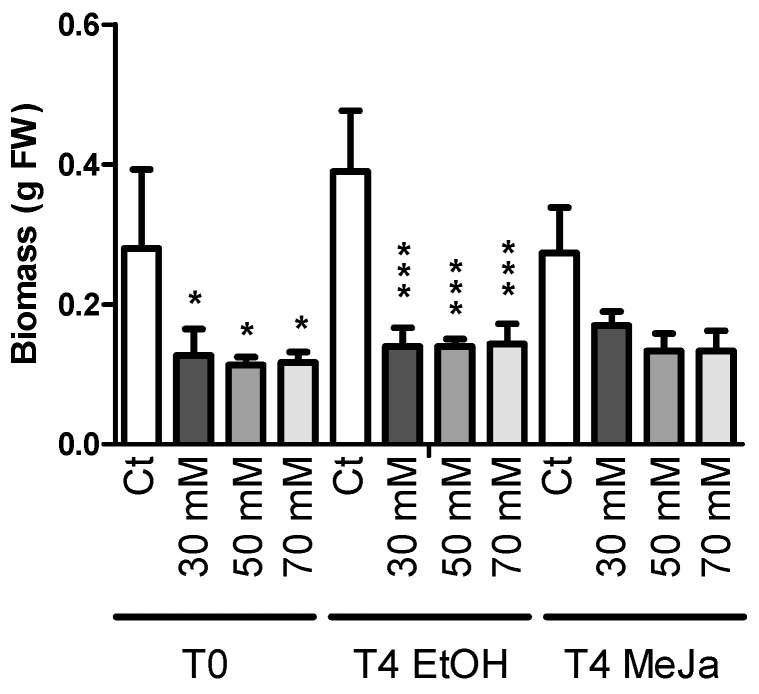
Effect of eliciting treatments on the biomass. The tested MCD concentrations were 0, 30, 50 and 70 mM. One hundred micromoles of MeJA and EtOH 0.1% were applied after 18 days of culture and for four days. Each point represents the mean with standard deviation of three biological replicates. Each value was compared to its corresponding control using ANOVA test (* *p* < 0.5, *** *p* < 0.001). Biomasses of different controls were not statistically different.

**Figure 8:**
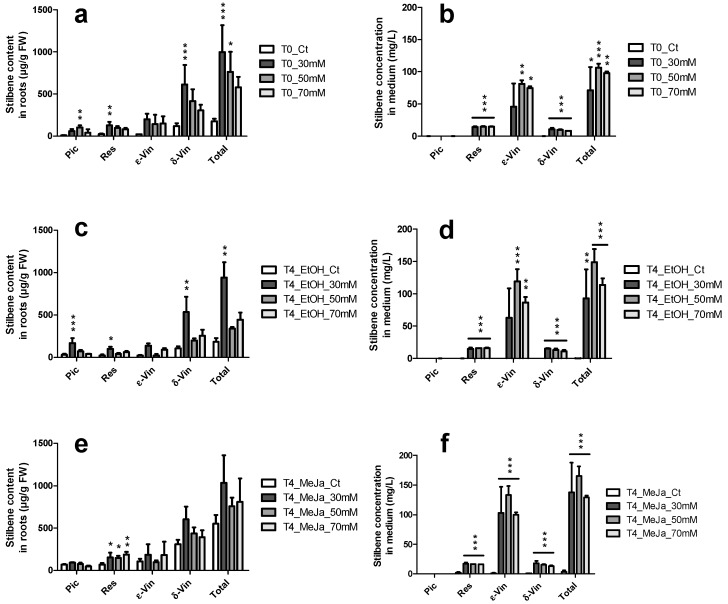
Amounts of stilbenes produced in response to treatments with MCDs in the culture media over time. Stilbene measured in HRs and in the culture medium, respectively: (**a**,**b**) after 18 days of culture and before applying 100 µM MeJA or EtOH; (**c**,**d**) four days after applying EtOH; and (**e**,**f**) four days after applying MeJA at 100 µM. Each point represents the mean with standard deviation of three biological replicates. Pic: piceid; Res: resveratrol; ε-vin: ε-viniferin; δ-vin: δ-viniferin. Each value was compared to its corresponding control using ANOVA test (* *p* < 0.5, ** *p* < 0.01, *** *p* < 0.001).

Cyclodextrins (CDs) and particularly methyl-β-CDs (MCDs) have also been described as strong inducers of stilbene production in grapevine cell suspensions [49]. They have been reported to display a synergistic effect when used in combination with MeJA [35,41,50,51,52,53]. However, their effect on the production of stilbenes in grapevine HRs is unknown as yet. The effect of CAVASOL^®^ W-7 MCDs alone or in combination with MeJA was evaluated on both root growth (Figure 7) and stilbene content (Figure 8). UPLC chromatograms are displayed in Figure S3.

CD concentrations used were chosen according to previous reports showing high stilbene production levels using grapevine cell suspensions [51,54,55]. Due to the high MCD concentrations applied, these were prepared directly in the culture medium before autoclaving. The HRs were thus cultivated with 0, 30, 50 or 70 mM MCD for 18 days before treatment with MeJA. Measurements were made four days after the application of MeJA. Figure 7 shows a strong effect of MCDs on HR growth at concentrations of 30, 50 and 70 mM, with almost no growth during the first culture phase of 18 days. MeJA treatment inhibited the growth of roots without MCDs but had no additional effect when applied on cultures containing MCDs. Previous results obtained for grapevine cell suspensions treated with 50 mM MCDs described a detrimental effect of the latter alone on cell growth [51]. MeJA treatment is known to have an even stronger inhibitory effect on the growth of grapevine cell suspension cultures [18,19,39,42,56]. Similar observations were made on HR cultures of *Scutellaria lateriflora* [57]. The use of MCDs on HRs has been reported to enhance the production of other phenolic compounds in *S. lateriflora* and to induce the production of stilbenes including resveratrol and derivatives such as prenylated arachidin-1 and arachidin-3 in peanut hairy root cultures [22].

Different elicitors were tested on those cultures, including sodium acetate, H_2_O_2_ as well as MeJA, CD or the combination of both. The best stilbene production in this system was achieved with 100 µM MeJA in combination with 9 g/L MCD, reaching 249 mg/L stilbenes excreted in the culture medium with a majority of arachidin-1 and arachidin-3. The use of MCDs on grapevine HR cultures displays a large induction effect on stilbenes accumulated in roots and excreted into the medium (Figure 8). After 18 days of culture, a clear increase in all quantified stilbenes in roots treated with MCDs was observed: control roots showed intracellular concentrations of 175 µg/g FW. In contrast, MCD-treated roots contained 999, 762 and 580 µg stilbenes/g FW before MeJA treatment for 30, 50 and 70 mM MCDs, respectively. The concentrations of stilbenes measured in the culture medium were 0.1 mg/L for the control and 71, 106, and 98 mg/L for the same CD-treated cultures. These concentrations represent an increase of stilbene production in comparison to control of 5.7, 4.4 and 3.3 folds in the roots and of 711, 1060 and 980 folds in the culture medium (Figure S4).

**Figure 9:**
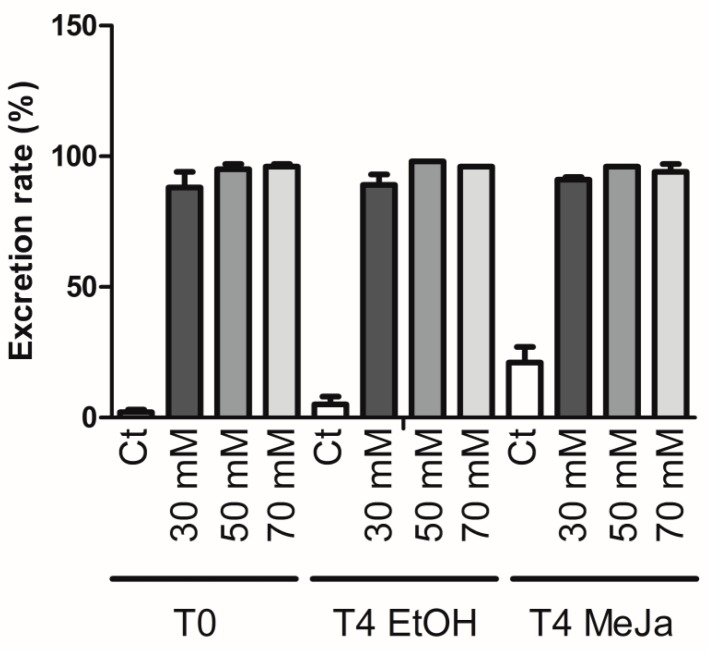
Excreted stilbenes as a proportion of total stilbenes in response to MeJA, MCDs and in combination. Each point represents the mean with standard deviation of three biological replicates.

The MeJA treatment was applied after 18 days of culture and its effect was measured four days afterwards. Addition of 100 µM MeJA induced further accumulation of stilbenes in the roots and the culture medium (Figure 8), as previously reported in grapevine cell suspensions [18,19]. Control roots and medium contained 183 µg/g FW and 0.3 mg/L stilbenes, respectively, after four days. A maximum production of stilbenes in roots was observed for roots treated with 30 mM MCDs and 100 µM MeJA, reaching up to 1034 µg/g FW total stilbenes after four days, which represents a 5.6-fold increase in production. In the medium, the highest stilbene concentrations were observed in the cultures treated with 50 mM MCDs and 100 µM MeJA, which yielded up to 165 mg/L total stilbenes after four days, corresponding to an increase of 571 folds in excreted stilbenes. The measured rates of excreted stilbenes show extremely high values of more than 90% of total stilbenes for all three tested MCD concentrations (Figure 9). δ-viniferin was slightly less excreted, with 80% found in the extracellular medium and piceid which remains mostly intracellular (Figure S3b).

These data support the fact that, besides their well-known eliciting effects on stilbene production, MCDs facilitate the outflow of stilbenes by increasing their solubility in the medium [58]. Although 30 mM MCDs leads to the highest stilbene content in roots, a dose of 50 mM is more suitable due the high quantity of excreted stilbenes obtained. In fact, high yields of excreted stilbenes facilitate their extraction from the cultures. Production of resveratrol reaching up to 5 g/L has been reported using MCDs in grapevine cell suspensions [59]. Although the use of HRs presents unique advantages, this system still needs further optimization in terms of growth conditions to reach similar stilbene concentrations. For example, the biomass reported here with HRs was 8 g FW/L after 18 days of culture with MCDs. In comparison, grapevine cell suspensions can reach up to 500 g FW/L [19]. Various approaches could be used to improve growth and thus biomass production before elicitation to reduce this limitation. A thorough optimization of the culture medium, by identifying limiting nutrients and potential growth factors, could lead to an improvement of growth. In the same way, growth conditions can be modified to optimize gas transfers, by shaking or changing the volume of the cultures. Comparing specific stilbene production in grapevine cell suspensions shows a total stilbene production of 13.4 mg/g FW [53], whereas it reached 18.1 mg/g FW in grapevine HRs, demonstrating that this system could represent a promising tool for stilbene production.

## 3. Materials and Methods

### 3.1. Plant and Bacterial Materials

Six-week-old in vitro plantlets from *Vitis vinifera* cv Pinot Noir 40024 were used for the transformation experiments. They were cultivated in a 16 h/8 h day/night photoperiod on McCown Woody Plant Medium (Dushefa, Haarlem, The Netherlands). All plant cultures were kept at 23 °C.

Two bacterial strains were tested: *Rhizobium rhizogenes* strain ATCC 15834 and *Rhizobium rhizogenes* ICPB TR7. The cultures were initiated in 10 mL Yeast Mannitol Broth YMB medium [60] for 24 h at 28 °C before being pelleted and resuspended in 20 mL in the corresponding plant medium supplemented with 1 mg/L 2,4-dichlorophenoxyacetic acid (2,4-D). 2,4-D was used to induce a short dedifferentiation to facilitate transformation [31,32]. The explants tested consisted of entire leaves with petioles or 1-cm-long sections of the stem. For plant infection, three different methods were compared: (1) wounding explants with a scalpel previously infected with the bacterial suspension; (2) immersing wounded explants in the bacterial suspension for 5 min; and (3) directly pricking 24 in vitro plantlets with an infected needle on 3 spots of the stem.

After infection, explants were transferred onto solid B5 medium [61] and co-cultivated in darkness. After 48 h, the explants were rinsed in B5 + 600 mg/L cefotaxime and cultured on solid B5 medium 3% sucrose supplemented with 300 mg/L cefotaxime. When the first roots appeared on the explants, and measured 1 cm or more, they were isolated and cultured on B5 medium + 300 mg/L cefotaxime. They were grown for 3 months on solid B5 medium + 300 mg/L cefotaxime and subcultured every 3 weeks, then transferred to liquid B5 medium + 300 mg/L cefotaxime. Each line was routinely subcultured every 40 days and the medium was changed every 10 days. The antibiotic pressure was removed 4 months after isolation, once the absence of *Rhizobacteria* was confirmed by PCR.

### 3.2. Confirmation of the Genetic Transformation

The status of genetically transformed roots was verified by PCR, by searching for the *rolC* transgene and the absence of bacterial *virD2.* Primer characteristics are described in Table 1 and sequences were taken from data available in the literature [62,63]. Specific amplification was achieved using Firepol^®^ polymerase (Solis Biodyne) with a thermal cycler (Progene, Thechne): 12 min activation at 95 °C, followed by 35 cycles of 30 s at 95 °C, 30 s at 56 °C and 45 s at 72 °C, and a final 2 min extension time at 72 °C. The bacterial strain was used as a double positive control, a wild type *Escherichia coli* strain was used as a double negative control and *Brassica rapa* hairy roots were used as a transformation-positive control [64].

### 3.3.Improvement of Growth Conditions

The best medium and sucrose concentrations tested were determined in non-limiting conditions. Each culture was started in 100-mL flasks, containing 20 mL medium and one primary root tip of approximately 2 cm. They were cultivated at 110 rpm in darkness for 35 days. Weight measurements and medium renewal, to avoid excessive medium loss over time, were carried out every 3 or 4 days. All tests were done in triplicate.

The tested media were MS [65], B5 [61], MC which is used to cultivate *Vitis vinifera* cv Pinot Noir in vitro plantlets [66] and SH [67] as well as 1/2 MS, 1/2 B5, 1/2 MC and 1/2 SH containing 3% sucrose. The different sucrose concentrations tested were 1%, 2%, 3%, 4%, 5% and 6% in B5 medium.

The growth rate µ was determined from the exponential trend line of the mean growth curve, with biomass equation X = X_0_ e ^µ(t−T0)^. The doubling time Td was calculated using $Td=\frac{\ln\left(2\right)}{µ}$ [68].

### 3.4. Growth Kinetics

The growth kinetic parameters were calculated from cultures in 100-mL flasks, containing 20 mL 1/2 SH medium with 2% sucrose, inoculated with two primary root tips of approximately 2 cm. Three flasks were used for each point to determine the fresh weight. Roots and media were stored at −20 °C for stilbene quantification. The growth rate µ was calculated using the slope of the curve given by mean values of triplicates.

### 3.5. Stilbene Extraction

Stilbenes were extracted from the medium as described in [69,70]. Liquid–liquid extractions using 10 mL ethyl acetate for 10 mL medium were carried out. Ethyl acetate phases were then collected, evaporated in a rotavapor (Laborota 4000-Efficient, Heidolph with PC3001 VARIO vacuum pump, Vacuubrand, Schwabach, Germany) and the extracts were resuspended in 1 mL methanol (MeOH) for UPLC quantification.

Stilbene extraction from roots was adapted from [69,70] using roots ground in liquid nitrogen. One hundred micrograms was weighed and extracted with 1mL 85% MeOH for 2 h in a thermoshaker at 850 rpm, then centrifuged. The supernatant was collected in a glass tube and the pellet was resuspended in 1 mL 100% MeOH for 1 h with a thermomixer (Eppendorf) at 850 rpm, and then centrifuged. The supernatants were pooled, evaporated in a Vacuum Concentrator 5301 (Eppendorf) and resuspended in 1 mL MeOH for UPLC quantification.

### 3.6. UPLC Analysis

UPLC analyses were done using an Acquity UPLC (Waters, Guyancourt, France) system, with a C18 Cortecs^®^ (Waters) column (2.1 × 100 mm, particles: 1.6 µm, pore size: 90 Å), maintained at 30 °C. The separation conditions were taken from [68]. Briefly, a gradient at 0.5 mL/min starting from 90% H_2_O +0.1% formic acid (A) and 10% acetonitrile +0.1% formic acid(B) to 40% A and 60% B, followed by 5 min rinsing was used. Stilbene fluorescence was measured with an Aqcuity fluorometer (Waters) with an excitation wavelength of 330 nm and an emission wavelength at 375 nm. Piceid (3-*O*-β-d-resveratrol glucoside), resveratrol (3,5,4′-trihydroxystilbene), δ- and ε-viniferins (resveratrol dehydrodimers), in both their *cis* and *trans* forms, were identified and quantified as described previously and used as external standards [71,72]. Resveratrol, piceid and ε-viniferin were purchased from Sigma at the time of the experiments. Supplementary data concerning stilbene identification are available in [73]. Quantification of piceid and resveratrol was expressed as *trans*-resveratrol equivalents and that of δ and ε-viniferins as *trans*-ε-viniferin equivalents. All extractions were done in subdue light to limit isomerization of *trans* stilbenes to the *cis* forms. The concentrations shown are the sum of *cis* and *trans* forms measured. Total stilbenes represent the sum of these stilbenes.

### 3.7. Induction Treatments

Methyl jasmonate (MeJA) (Sigma Aldrich, St. Quentin Fallavier, France) was added to the culture medium (1/2 SH supplemented with 2% sucrose) from an ethanol (EtOH) solution after the exponential growth phase, corresponding to 18 days of culture. Final concentrations at 100 µM and 200 µM were chosen, with a maximum concentration of 0.1% EtOH in the medium. Controls were obtained by addition of EtOH to a final concentration of 0.1%. All experiments were conducted in triplicate.

For the MeJA and CD experiments, CAVASOL^®^ W-7 MCDs (WackerChemie AG, Burghausen, Germany) were dissolved in the culture medium (1/2 SH supplemented with 2% sucrose) to final concentrations of 30 mM, 50 mM and 70 mM before autoclaving and MeJA was added after 18 days at a final concentration of 100 µM.

## 4. Conclusions

Hairy root systems are particularly attractive as study and bioproduction tools due to their specific properties such as high genetic and phytochemical stability, good growth rates and their highly controlled environment.

In the present work, the growth and phenotype of grapevine cv Pinot Noir HRs has been reported. Moreover, their ability to respond to elicitation treatments that lead to the significant production and excretion of stilbenes into the culture medium has been demonstrated. The results show that grapevine hairy root cultures are able to provide high yields of secreted stilbenes, particularly ε-viniferin, when treated with methyl jasmonate and MCDs. This makes them a good candidate for the production of highly pure stilbenes. The up-scaling of such systems has already been proven feasible [74] and achieved by different companies (10,000 L *Panax* bioreactor) [75].

## Figures and Tables

**Table 1:** Sequences of primers used for confirmation of the genetic transformation [61,63].

Primer	Direction	Sequence
***rolC***	Forward	5′-ATGGCTGAAGACGACCTGTG-3′
	Reverse	5′-TAGCCGATTGCAAACTTGCAC-3′
***virD2***	Forward	5′-ATGCCCGATCGAGCTCAAG-3′
	Reverse	5′-GACCCAAACATCTCGGCTG-3′

## Supplementary Materials

Supplementary materials can be accessed at: http://www.mdpi.com/1420-3049/21/12/1703/s1.

## Abbreviations

The following abbreviations are used in this manuscript:

B5	Gamborg medium
CD	Cyclodextrin
HR	Hairy roots
JA	Jasmonate
MeJA	Methyl jasmonate
MS	Murashige and Skoog medium
SH	Schenk and Hildebrandt medium
