# Supplementary material for: Enhanced Stilbene Production and Excretion in Vitis vinifera cv Pinot Noir Hairy Root Cultures
Source: Molecules. 2016 Dec 10;21(12):1703. doi: 10.3390/molecules21121703 (PMC6273728; doi:10.3390/molecules21121703)
Supplement: Supplementary file 1 [file molecules-21-01703-s001.pdf]

Supplementary Materials: Enhanced Stilbene Production and Excretion in *Vitis vinifera* cv Pinot Noir Hairy Root Cultures

Leo-Paul Tisserant, Aziz Aziz, Nathalie Jullian, Philippe Jeandet, Christophe Clément, Eric Courot and Michèle Boitel-Conti

1. Confirmation of the Transformation

The transformation of the selected hairy root lines was done by verification of the presence of the auxin-synthesis coding *rolC* gene and the absence of the virulence *virD2* bacterial gene. As shown in Figure S1; all 9 selected lines were confirmed to be transformed.

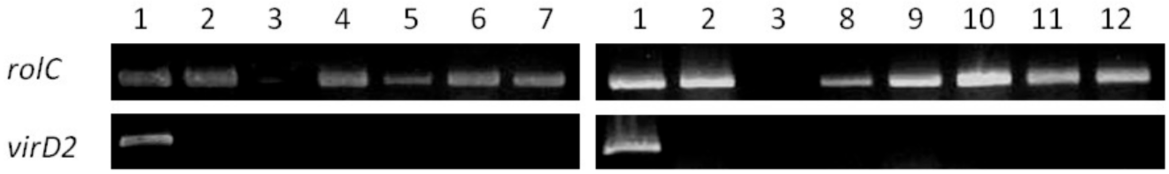

Figure S1. PCR confirmation of the transformation. (**1**) *R. rhizogenes* ATCC 15834 double positive control; (**2**) *Brassica rapa* hairy roots as a *rolC* positive control; (**3**) *Vitis vinifera* in vitro plantlet roots as double negative control. (**4**) Root line 19A; (**5**): Root line 16C; (**6**) Root line 18B; (**7**) Root line 13F; (**8**) Root line 19B; (**9**) Root line 16D; (**10**) Root line 3A; (**11**) Root line 13G; (**12**): Root line 28C.

2. Proportion of Different Excreted Stilbenes

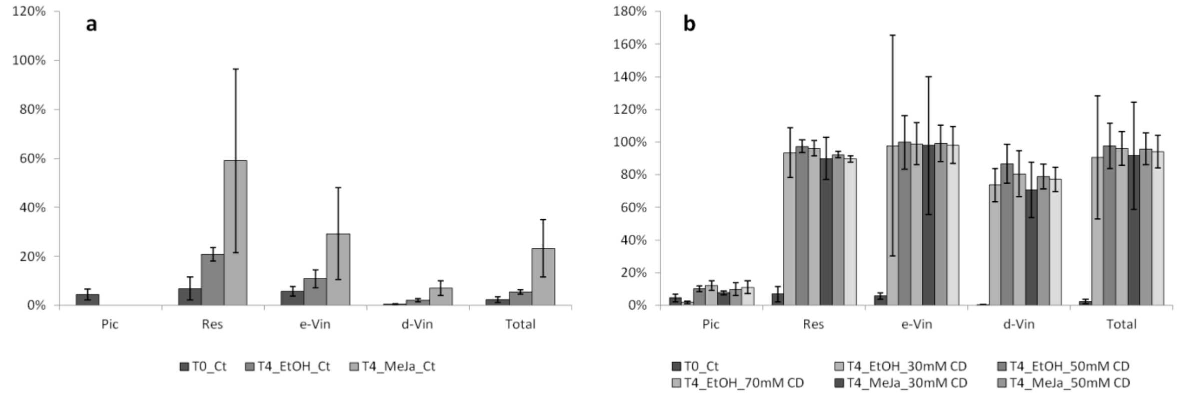

Figure S2. Proportion of each stilbene excreted in the culture medium over the sum of their intracellular and excreted amounts.

3. UPLC Chromatograms

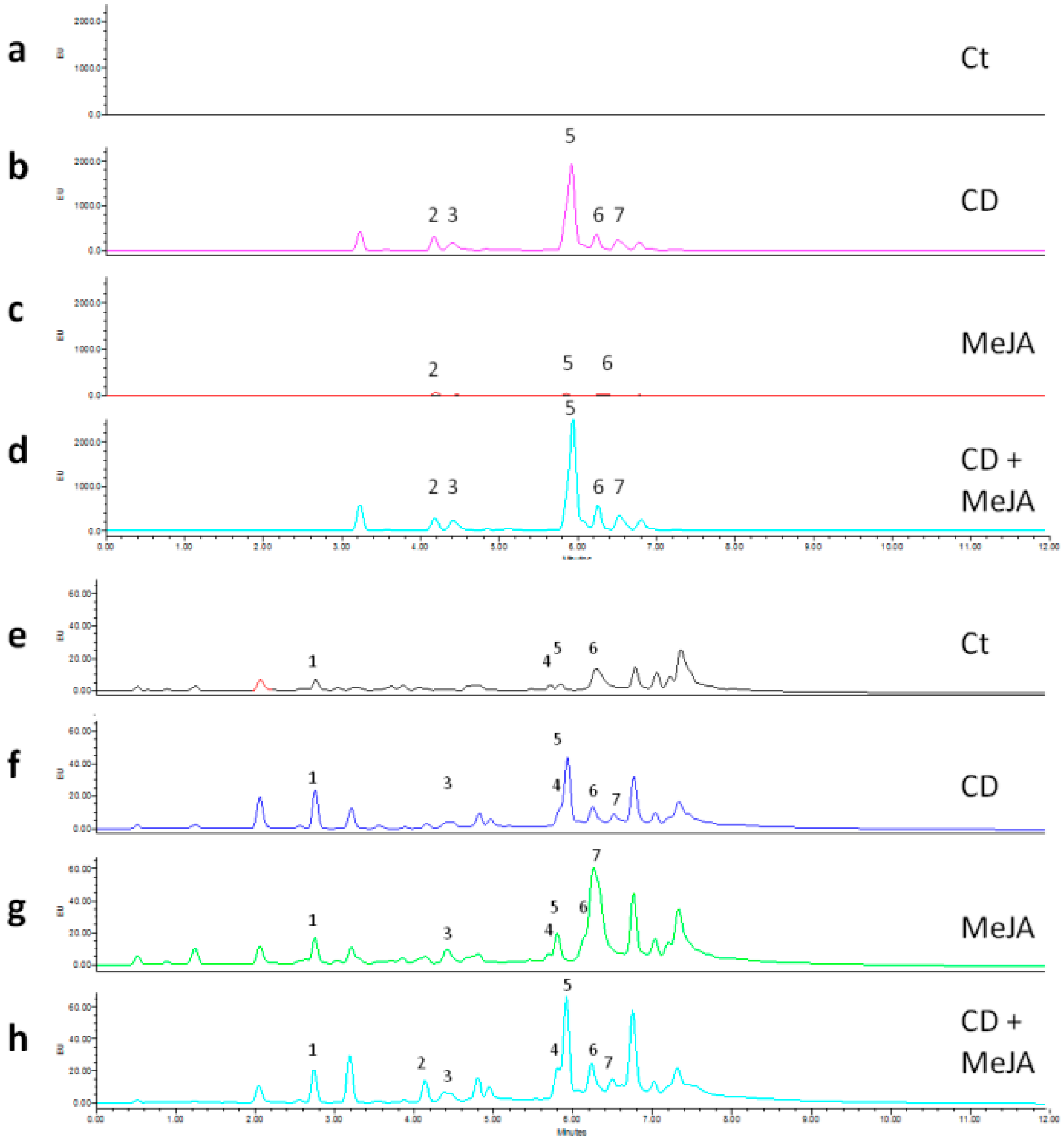

Figure S3. Examples of UPLC chromatograms for culture medium extracts (**a**–**d**) and root extracts 96 h after elicitation (**d**–**h**); for controls; 50 mM MCD; 100 µM MeJA; and 100 µM MeJA in combination with 50 mM MCD respectively. 1: t-piceid; 2: t-resveratrol; 3: c-resveratrol; 4: c- ε-viniferin; 5: t-ε-viniferin; 6: t-δ-viniferin; 7: c-δ-viniferin.

4. Fold-Increase in Stilbene Production in Comparison to Controls

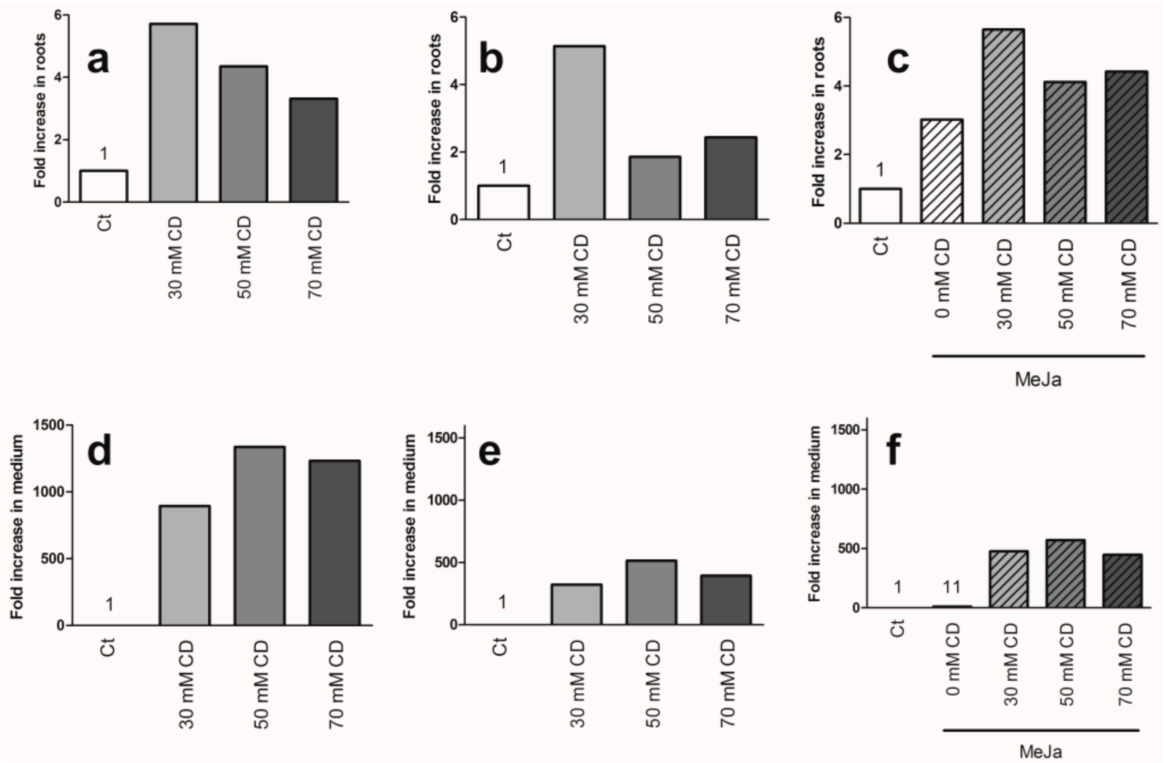

Figure S4. Relative amounts of total stilbenes quantified for each treatment in comparison to control. MCD concentrations tested were 0; 30; 50 and 70 mM. (**a**–**c**) Fold-increase in production of stilbenes in roots in comparison to control before induction; after 4 days without MeJA and after 4 days with 100 μM MeJA respectively; (**d**–**f**) Fold-increase in production of stilbenes in the culture medium in comparison to control before induction; after 4 days without MeJA and after 4 days with 100 μM MeJA respectively.
